# The Use of Intralipid Infusions in the Prevention of Embryo Implantation Failure

**DOI:** 10.7759/cureus.53368

**Published:** 2024-02-01

**Authors:** Okorie C Anya, Eniola R Ajayi, Henry R Solanke, Adaeze I Ohanaka, Kokei D Ubana

**Affiliations:** 1 Reproductive Medicine, Primecare Fertility Clinic, Abuja, NGA; 2 Public Health, Central Michigan University College of Medicine, Michigan, USA; 3 Food Safety and Quality Assurance, Sensei Ag, Aldergrove, CAN; 4 Obstetrics and Gynaecology, South Qunfudah General Hospital, Jizan, SAU; 5 General Practice, Nakhal Health Center, Nakhal, OMN

**Keywords:** uterine natural killer cells, recurrent pregnancy loss, intravenous lipid emulsion, in vitro fertilization, intralipid, immunomodulation

## Abstract

Intralipids have been suggested to suppress uterine natural killer cell activity, which could potentially improve implantation rates in women with recurrent loss. We report a case of a 41-year-old African woman with recurrent pregnancy loss who had elevated uterine killer cell activity and for whom intralipid infusion was used to achieve pregnancy. We recommend routine uterine natural killer cell testing for women with recurrent pregnancy loss and further research on newer intravenous lipid emulsions in fertility medicine.

## Introduction

Intralipid is an intravenous lipid emulsion invented by a Swedish physician and nutrition researcher called Arvid Wretlind, and it was approved for clinical use in Sweden in 1962 [1]. Intralipid is an intravenous lipid emulsion containing 20g of soyabean oil, 1.2g of phospholipids, 2.25g of glycerin, and water for injection.

One of the early indications for the use of intralipid in clinical practice was as a source of parenteral nutrition in intensive care patients. It was indicated in patients who could not tolerate enteral feeding, or if enteral nutrition was not sufficient to meet target caloric intake, and also in those who had suppression of gastrointestinal activity e.g., immediately following injury or surgery [2]. Many critically ill patients admitted to the intensive care unit (ICU) usually enter a state of negative energy balance during the first few days following admission, and this energy deficit often progresses during their ICU stay and may result in malnutrition and adverse outcomes like such as an increased risk of sepsis [3]. Intralipids have demonstrated efficacy and safety in delivering vital nutrition to critically ill patients and averting these negative outcomes. However, the newer intravenous lipid emulsions that utilize partial substitution of soybean oil with olive oil, fish oil, and medium-chain triglycerides either alone or in combination, have demonstrated potential benefits in terms of reduced impacts on oxidative stress and differential effects on cell-mediated immunity and inflammation [4].

Before arriving at the medication rack of the fertility clinics, intralipids were employed in the emergency department, where they were used in the management of local anesthesia systemic toxicity (LAST). In 1997, Weinberg et al. [5] pretreated rats with bupivacaine-induced cardiac arrest with an intravenous lipid infusion and found that the time to cardiac arrest was prolonged and the recovery time was quicker. As a result, intralipid infusion became known not only as a parenteral nutrition option but also as a treatment for LAST. One of the proposed mechanisms is the lipid shuttle theory where the lipid emulsion removes the lipophilic local anesthetic from the heart and brain, where it causes the most severe damage, and redistributes it to the liver and muscle tissues for acute detoxification and digestion [6]. 

In the fertility clinic, one of the factors that can lead to implantation failure of apparently reproductively competent embryos is dysfunction of the immune system. Although a controversial topic of discussion, it has been found that the number and/or activity of the uterine natural killer (NK) cells peaks during the mid-secretory phase of the menstrual cycle, which also coincides with the embryo implantation period, and might be responsible for implantation failure [7]. This hypothesis led to the proposed therapy that aims to alter the immune milieu in an effort to improve outcomes with the use of intralipids, intravenous immunoglobulin corticosteroids, anti-tumor necrosis factor (TNF), and granulocyte-colony stimulating factor (GSF) [8].

This report describes the case of a 41-year-old African woman with a background history of four early pregnancy losses within a five-year period of marriage. She had two myomectomies prior to embarking on in vitro fertilization (IVF) treatment, and prior to commencing her second IVF cycle, she did a uterine NK cell activity test which detected high NK cell activity in her endometrium. This led to the use of intralipids during her second IVF cycle, based on recommendation, and the eventual achievement of a viable clinical pregnancy with three gestational sacs.

## Case presentation

We report a case of a 41-year-old female patient who first presented to our fertility clinic, which is a private fertility clinic located in Abuja, Nigeria, on July 13, 2022, for IVF treatment. She had been married for about five years at that time and had not been able to have a child. At the index consultation, there were no current symptoms or complaints, and her menstrual cycle was still fairly regular.

Her gynecological history revealed that she had suffered five episodes of early pregnancy loss within the past five years, most of them occurring between six and eight weeks of gestation, and none was occasioned by retained products of conception that required evacuation. She had a history of multiple uterine fibroids in the past and had had two myomectomies, the first in 2011 and the second in 2020. She had no underlying medical conditions and routinely used folic acid and dehydroepiandrostenedione (DHEA) tablets to improve her ovarian reserve. Her family and social history were non-contributory.

Prior to commencing her IVF treatment, she did a fertility work-up, which according to our local protocol includes: urine analysis, microscopy, culture, and sensitivity, blood group and genotype, fasting blood sugar, a complete blood count, a renal and liver function test, a venereal disease research laboratory (VDRL) for syphilis, hepatitis B surface antigen (HbsAg), hepatitis C serology, human immunodeficiency virus (HIV) serology, anti-Müllerian hormone (AMH), serum prolactin level, and thyroid function test. For her partner, the following tests were conducted: seminal fluid analysis (SFA), blood group and genotype, hepatitis B surface antigen (HbsAg), hepatitis C serology, and human immunodeficiency virus (HIV) serology.

Significant results from the investigations were low AMH levels, slightly elevated serum prolactin levels for the patient, and an AC genotype for her partner. Based on the above findings, she was commenced on cabergoline tablets at a dose of 0.5 mg twice weekly for six weeks, and an 'agonist long-protocol' IVF treatment was planned for her, which was to commence with her September 2022 cycle.

Her next relevant menstrual period commenced on September 22, 2022, and on October 12, 2022, which was Day 21 of her cycle, the treatment protocol was commenced. She received 3.75 mg of an intramuscular injection of leuprolide, a gonadotropin-releasing hormone agonist (GnRH agonist) as well as fertility supplements and antioxidants. She had a withdrawal bleed on October 26, 2022, and was then commenced on gonadotropins for ovarian stimulation on October 27, 2023, with 450 IU of human menotropin gonadotropin (HMG), which is a combination of follicle-stimulating hormone and luteinizing hormone. This was given for four days, and on Day 6, it was reduced to 300 IU. By Day 14, she received 5,000 IU of beta-human chorionic gonadotropin (HCG) injection, and on November 8, 2022, her oocytes were retrieved in the embryology theater.

The retrieved eggs were fertilized with her partner’s sperm cells through intra-cytoplasmic sperm cell injection (ICSI) and incubated for five days. By the fifth day, three blastocysts were transferred into her endometrium on November 11, 2022, and a serum pregnancy test was done on November 25, 2022, which came out negative. A ‘failed IVF review consultation’ was done, and she was recommended to do a series of immunological tests to rule out immunologic causes of implantation failure. This decision was made based on her history of recurrent pregnancy losses.

The patient opted to do her immunology tests abroad and did the following: anti-phospholipid antibodies, anti-nuclear antibodies, total T lymphocyte count, and total NK cell count and activity. The results of these tests are represented below (Tables 1-4).

**Table 1:** Results of lymphocyte sub-population test NK cells: natural killer cells

Lymphocyte subpopulations	CD marker	Result	Normal values (%)
Total T-Lymphocytes	СD3+	72.0	67-76
Subpopulation of NK cells	NK cells	12.0	2-13
Subpopulation of NK cells	Endometrial NK cells	14.2	3-13

**Table 2:** Results of NK cell activity test NK cells: natural killer cells

Marker	Result	Normal value (%)
NK cell activity (NKa)	13.0	N < 10%
NKa in the presence of IVIg	9.2	10% expected decrease of the previously determined NKa in the absence of IVIg
NKa in the presence of Intralipid	9.0	

**Table 3:** Results of anti-phospholipid antibody testing

Anti-phospholipid antibodies	Result	Reference range
Antiphospholipid screening	4.2 (Neg)	(Normal <5; borderline 5-8; positive >8 IU/ml)
Anticardiolipin screening	5.1 (Neg)	(Normal <10.0; positive ≥10.0 U/ml)
Anticardiolipin IgM	4.8 (Neg)	(Normal <7.0; positive ≥7.0 U/ml)
Anti -β2GPI screening	3.9 (Neg)	(Normal <10.0; positive ≥10.0 U/ml)
Anti -β2GPI - IgM	2.7 (Neg)	(Normal <5; borderline 5-8; positive >8 IU/ml)
Anti-prothrombin screening	11.9 (Neg)	Normal <20 U/ml
Anti-prothrombin IgM	3.8 (Neg)	(Normal <5; borderline 5-8; positive >8 IU/ml)
Anti-annexin V IgG	1.2 (Neg)	(Normal <5; borderline 5-8; positive >8 IU/ml)
Anti-annexin V IgM	1.4 (Neg)	(Normal <5; borderline 5-8; positive >8 IU/ml)

**Table 4:** Results of anti-nuclear antibody screening test

Anti-nuclear antibody	Result	Reference range
Antinuclear antibody screening	1.1 (Borderline)	Normal <1 IU/ml; borderline 1 -1.2 IU/ml; positive >1.2 IU/ml

Her second IVF cycle began with an ‘agonist long protocol’ on the 21st day of her February 2023 cycle. She got an intramuscular injection of 3.75 mg of leuprolide and also began taking antioxidant tablets daily as well as low-dose acetylsalicylic acid. Progesterone tablets were also given at 5 mg twice a day for seven days. After the progesterone tablets, her withdrawal bleeding occurred on March 22, 2023, and ovarian stimulation was commenced the next day (Day 2) with 450 IU of HMG. By her sixth day, the HMG dose was reduced to 300 IU. She had serial trans-vaginal ultrasound scans on Days 2, 6, 9, and 12, and her Day 12 scan revealed that several follicles on both ovaries had attained an average size of 17 mm. This led to the administration of 5,000 IU of beta-human chorionic gonadotropin (HCG) intramuscularly to mimic the ‘luteinizing hormone (LH) surge’. By Day 14 of her cycle, she underwent an oocyte retrieval procedure, after which she was commenced on daily intramuscular injections of 100 mg progesterone, which were to be continued for the next six days. On that same day (Day 14), she received 500 ml of 20% intralipid infusion intravenously, which she tolerated well. Her eggs were fertilized with her partner’s sperm cells via ICSI, and four blastocysts were transferred into her endometrium, 1.5 cm from the fundus, on April 9, 2023, being the 19th day of her cycle. Three days after her blastocyst transfer, she received the second dose of 500 ml of 20% intralipid infusion, which was administered intravenously, and no adverse effects were encountered.

Two weeks after her blastocyst transfer, a serum pregnancy test was done, which came out positive. On May 8, 2023, she did a transvaginal ultrasound scan to confirm clinical pregnancy, and three gestational sacs with fetal poles, yolk sacs, and good cardiac activity were detected. She is currently in the third trimester of her pregnancy, with an estimated delivery date of December 27, 2023.

Online follow-up consultations revealed that she has been regular with her antenatal visits, routine vaccinations, and medications, and both the patient and her babies are in a good state of health.

## Discussion

For successful embryo implantation to occur, there must be maternal-fetal immune tolerance, and this process involves some immune cells and their various molecules. As the trophoblast of the embryo invades the endometrium, local immune cells at the implantation site are activated by the fetal antigens, which include innate lymphocytes, T cells, decidual dendritic cells, and macrophages [9]. These cells are responsible for mediating maternal-fetal tolerance and promoting placental development, but immune dysregulations can also occur, leading to adverse pregnancy outcomes [9]. Amongst the immune cells that are involved in maternal-fetal immune tolerance, the uterine NK cells, which constitute between 70% and 90% of the lymphocytes found in the uterus, have been extensively studied regarding their association with reproductive success or failure [7].

There have been contradictory conclusions within several studies regarding the relationship between the number of uterine NK cells in the endometrium and the prediction of subsequent pregnancy outcomes. One major study was a comprehensive review done by Konstantinos et al. and published in August 2021, which demonstrated by numerous studies an association between uterine NK cells and recurrent implantation failure or recurrent miscarriages [10]. The same study also showed a lack of connection between uterine NK cells and reproductive failure [10]. There are some theories of how these NK cells led to negative pregnancy outcomes, and one of the most popular of them is that an excessive number of uterine NK cells could lead to increased peri-implantation blood flow and excessive oxidative stress to trophoblast cells, thereby increasing the risk of pregnancy loss [11].

Despite the absence of clear-cut evidence that there is a causative relationship between increased number or activity of uterine NK cells and early pregnancy loss and no documented recommendation from clinical governing bodies, such as the Human Fertilization and Embryology Authority, adjuvant therapies are still being offered to women undergoing IVF or attending recurrent miscarriage clinics based on the 'myth' that uterine NK cells need suppressing to prevent embryo implantation failure [8]. This led to the use of immunotherapy to improve the clinical pregnancy rate during IVF, and the most common of them include the administration of oral corticosteroids, in particular prednisolone, intralipid infusion, and intravenous immunoglobulin [10]. One of the earliest studies that focused on the use of intralipid infusions for suppressing NK cell activity was one done by Roussev et al. [12], which was published in 2008. In this study, the aim was to establish the duration and efficacy of the suppressive effect of intralipids on NK cell functional activity. For the study, 50 women with abnormal NK cell activity (NKa) that was determined by flow cytometry using K562 cells as targets were recruited, and they received a 20% intravenous intralipid infusion. Results showed that 39 (78%) of them showed NKa suppression within the normal range within the first week and that this suppression lasted for about six to nine weeks [12]. Another recent one was a meta-analysis and systematic review done by Rimmer et al. [13] and published in 2021. It included five randomized clinical trials reporting on 843 women with an overall moderate risk of bias. All trials used 20% intravenous intralipid solution at the time of embryo transfer compared to 0.9% normal saline infusion or no intervention, and the results showed that the intravenous intralipid infusion group had a higher chance of clinical pregnancy (172 vs. 119, risk ratio 1.55, 95% confidence interval 1.16-2.07), post-treatment compared to no intervention [13].

These findings are the major reason for reporting this case because, out of the hundreds of patients seen with a history of embryo implantation failure both naturally and after IVF, this case was the only one that went as far as doing an NK cell activity test in a diagnostic center in Turkey. From her results, the anti-nuclear antibody titer was borderline high, the number of uterine NK cells was elevated, and their activity was also increased. These findings made her a good candidate for the use of intralipid infusion to observe its effect on NK cell activity and embryo implantation. She was eventually given two doses of 500 ml of 20% intralipid infusion on Days 14 and 22 of her IVF treatment cycle. Four weeks after her blastocyst transfer, clinical pregnancy was confirmed with a transvaginal ultrasound scan, which reported three gestational sacs with fetal poles, yolk sacs, and good cardiac activity. There are several protocols for the administration of intralipids during IVF treatment, many of which are center-based. In our center, the IVF intralipid protocol is to administer two doses of 500 ml of 20% intralipid infusion during the treatment cycle. The first dose is given on the day of oocyte retrieval, which is usually Day 14, for fresh embryo transfers (embryos formed by fertilization of the retrieved oocytes), and the second dose is on Day 22, which is usually three days after embryo transfer. For frozen embryo transfer (embryos that were formed much earlier and frozen for later transfer), the first dose of intralipids is given when the patient commences her daily intramuscular progesterone injections to prepare her endometrium for successful implantation. This is also usually around Day 14 of the treatment cycle. Our protocol has some similarities and differences with another popular protocol, which was reported by Ehrlich et al. [14] in a study that was published in 2019. The intralipid protocol for two IVF centers in Australia was reported. The first center, called IVF Australia (IVFA), administered two doses of 20% intralipid infusions; the first dose was within Days 5-9, and the second dose was after a positive pregnancy test using a beta-HCG value above 25 IU. The second center, called Fertility SA, also used a two-dose protocol; the first dose of intralipid was received on the oocyte retrieval day of a fresh embryo transfer cycle or on the embryo transfer day of a frozen embryo transfer cycle, and then the second dose was administered after a positive beta-HCG test [14].

There are some documented adverse effects associated with acute intravenous lipid emulsion administration, and they include acute kidney injury, acute lung injury, venous thromboembolism, hypersensitivity, fat embolism, pancreatitis, and even increased susceptibility to infection [15]. There is a paucity of data and studies that report adverse outcomes when using intralipids to increase implantation rates, and in fact, the study by Ehrlich et al. [14] reported a low rate of adverse events occurring during their study and also suggested that intralipids were safe immunomodulatory agents [14]. It was also mentioned in the same study that their finding was consistent with the findings in some other recent trials, which reported no adverse effects from using intralipids as an immunomodulator [14]. However, there were some studies that were done earlier that assessed the benefits and risks of the use of parental lipids in critical care patients in the intensive care unit (ICU). One of these studies reported that ICU patients usually have an increased production of reactive oxygen species and a decreased level of antioxidants, usually due to malnutrition, which leads to increased oxidative stress [4].

This oxidative stress led to lipid peroxidation and then protein and deoxyribonucleic acid (DNA) damage, which led to poorer outcomes in these ICU patients [4]. This study reported that replacing the polyunsaturated fatty acids (PUFA) obtained from soybean-based oils (such as the oils found in intralipids) with medium-chain triglycerides (MCT) or the mono-unsaturated fatty acids (MUFA) found in olive oil led to reduced production of lipid peroxides and cell damage, which occurred as a result of excessive oxidative stress [4]. What this finding means is that the MCT and MUFA were more resistant to oxidative stress than the PUFA found in soybean-based oils, which constitute 100% of the oils in intralipids. Although the mechanism of action of the intralipids in improving implantation rate is poorly understood, one popular theory is that they suppress the activity of uterine NK cells, which are responsible for increased oxidative stress around the trophoblastic cells of the embryo, which in turn prevents successful embryo implantation [11, 12]. So then, if MCTs and MUFA from olive oils are better candidates to resist the impact of oxidative stress, they may as well become better candidates for intravenous lipid emulsions (ILE) to use for the management of embryo implantation failure in order to improve the implantation rate and clinical pregnancy. There are newer commercially available intravenous lipid emulsions that contain olive oil and MCT, as opposed to the intralipids in which soybean oil constitutes 100% of the oil source, but there is a severe paucity of studies and data that investigate and report the efficacy of these newer ILEs in the management of embryo implantation failure.

Intralipids remain the most studied intravenous lipid emulsions, but with respect to their use in fertility medicine in the management of embryo implantation failure, there is insufficient evidence regarding their routine use, and a standard treatment protocol is also lacking [9]. A recent systematic review of the guidelines of the Royal College of Obstetricians and Gynecologists (RCOG), the American Society of Reproductive Medicine (ASRM), and the European Society of Human Reproduction and Embryology (ESHRE) concerning the diagnostics and treatment of recurrent pregnancy loss was done by Tomkiewicz et al. and published in July 2023 [16]. Reports from the review pointed out that it was suggested that uterine NK cells may have a significant role in trophoblastic invasion and angiogenesis in addition to being an important component of the local maternal immune response to pathogens, however, mention was made of one large study that checked the connection between uterine NK cell numbers and future pregnancy outcomes, and this study reported that raised uterine NK cell numbers in patients with recurrent pregnancy loss were not connected with an increased risk of miscarriage [17]. The review also pointed out that there is no unequivocal evidence of the impact of antinuclear antibodies (ANA) on recurrent pregnancy loss, and at the moment, no international guidelines recommend routine ANA testing or routine assessment of uterine NK cells in women with recurrent pregnancy loss [16].

## Conclusions

Currently, there are no recommendations from regulatory bodies that oversee reproductive issues and assisted conception for routine assessment of uterine NK cell number or activity in women with recurrent miscarriages, and the main reason for this is a lack of adequate evidence to prove a causative relationship between uterine NK cell number and activity and early pregnancy loss. With conflicting results regarding the effects of intralipids on NK cell activity, the only way out is for more research to be done in this domain. NK cell testing is highly unpopular in Nigeria as a whole, and this case was one out of thousands who had this test done. Having results that showed elevated uterine NK cell number and activity, the use of intralipid to observe its effect on implantation became imperative. With the occurrence of clinical pregnancy in this case, the decision to report it was solely to encourage routine testing for NK cell number and activity in women with a history of recurrent pregnancy loss or implantation failure, in order to add to the available data pool and provide more evidence. This can also be a driving factor for large-scale multicenter studies in Nigeria and Africa as a whole.

In the past 10 years, there has been the arrival of newer intravenous lipid emulsions (ILE), which have a different source of fatty acids. The intralipids have 100% soybean oil as their source of fatty acids, but these newer ILEs utilize medium-chain triglycerides, fish oil, olive oil, and some combinations of two or more oils. Intralipids are the most studied ILE, but some studies have also been done on these newer ILEs, mostly around their use in critically ill patients in the intensive care unit (ICU). Some of these studies have reported some advantages these newer ILEs have over intralipids in ICU settings, but there is a paucity of data on their application in fertility medicine. There is a possibility that these newer ILEs possess the “abilities” we have been searching for in the intralipids, and only by involving them in large-scale comparative studies and randomized clinical trials might we be able to make ground-breaking discoveries.
